# The oxidative demethylase ALKBH3 marks hyperactive gene promoters in human cancer cells

**DOI:** 10.1186/s13073-015-0180-0

**Published:** 2015-06-30

**Authors:** Robert Liefke, Indra M. Windhof-Jaidhauser, Jochen Gaedcke, Gabriela Salinas-Riester, Feizhen Wu, Michael Ghadimi, Sebastian Dango

**Affiliations:** University Medical Center, Department of General-, and Visceral Surgery, D-37075 Göttingen, Germany; Division of Newborn Medicine and Program in Epigenetics, Department of Medicine, Boston Children’s Hospital, Boston, MA 02115 USA; Department of Cell Biology, Harvard Medical School, Boston, MA 02115 USA; University Medical Center, Transcription Analysis Laboratory, D-37073 Göttingen, Germany; Epigenetics Laboratory, Institute of Biomedical Sciences, Fudan University, Shanghai, 200032 China

## Abstract

**Background:**

The oxidative DNA demethylase ALKBH3 targets single-stranded DNA (ssDNA) in order to perform DNA alkylation damage repair. ALKBH3 becomes upregulated during tumorigenesis and is necessary for proliferation. However, the underlying molecular mechanism remains to be understood.

**Methods:**

To further elucidate the function of ALKBH3 in cancer, we performed ChIP-seq to investigate the genomic binding pattern of endogenous ALKBH3 in PC3 prostate cancer cells coupled with microarray experiments to examine the expression effects of ALKBH3 depletion.

**Results:**

We demonstrate that ALKBH3 binds to transcription associated locations, such as places of promoter-proximal paused RNA polymerase II and enhancers. Strikingly, ALKBH3 strongly binds to the transcription initiation sites of a small number of highly active gene promoters. These promoters are characterized by high levels of transcriptional regulators, including transcription factors, the Mediator complex, cohesin, histone modifiers, and active histone marks. Gene expression analysis showed that ALKBH3 does not directly influence the transcription of its target genes, but its depletion induces an upregulation of ALKBH3 non-bound inflammatory genes.

**Conclusions:**

The genomic binding pattern of ALKBH3 revealed a putative novel hyperactive promoter type. Further, we propose that ALKBH3 is an intrinsic DNA repair protein that suppresses transcription associated DNA damage at highly expressed genes and thereby plays a role to maintain genomic integrity in ALKBH3-overexpressing cancer cells. These results raise the possibility that ALKBH3 may be a potential target for inhibiting cancer progression.

**Electronic supplementary material:**

The online version of this article (doi:10.1186/s13073-015-0180-0) contains supplementary material, which is available to authorized users.

## Background

Genomic DNA is continuously subjected to various harmful insults, such as UV light, ionizing radiation, or nucleic-acid modifying compounds, resulting in thousands of DNA alterations in each cell every day [1]. Such lesions can lead to DNA damage, which in turn favors mutagenesis, carcinogenesis, inflammation, and aging [2–5]. Accordingly, cells have multiple mechanisms to reverse damaging DNA modifications. In particular, DNA alkylation, a process of methylating specific nucleic acids, often requires repair to maintain genomic integrity. Alkylating agents are found ubiquitously in the environment, but DNA can also be alkylated as a natural by-product of cellular metabolism [6, 7]. For example, the universal methyl donor S-adenosylmethionine non-enzymatically methylates DNA [8, 9]. Alkylating agents preferentially attack single-stranded DNA (ssDNA) in the genome due to its higher accessibility [10–13], and some DNA modifications such as 1-methyladenine (1-meA) and 3-methylcytosine (3-meC) are primarily generated in ssDNA, because these positions are shielded in double-stranded DNA (dsDNA) [6].

DNA alkylation can be removed by base-excision repair (BER), direct reversal by methylguanine methyltransferase (MGMT), and dealkylation via the AlkB family [6, 7]. The AlkB enzymes belong to a large family of non-heme Fe(II) and 2-oxoglutarate-dependent dioxygenases, which catalyze numerous biological reactions, such as proline hydroxylation and histone demethylation [14]. AlkB was original discovered in *E. coli*, where it demethylates 1-methyladenine (1meA) and 3-methylcytosine (3meC) by oxidation of the N-linked methyl moiety. This reaction creates an unstable methyl-iminium intermediate that spontaneously hydrolyzes into formaldehyde and the non-alkylated base [15–17]. In mammalians, at least nine ALKB family members are known (ALKBH1-8 and FTO). DNA damage dealkylation reactions are mainly catalyzed by ALKBH2 and ALKBH3 [16]. Notably, ALKBH2 preferentially demethylates dsDNA while ALKBH3 demethylates ssDNA and RNA substrates, modified by 3-meC or 1-meA [16, 18, 19]. Since these modifications are predominantly generated in ssDNA and RNA, it has been proposed that the ALKBH3 repair function could be linked to transcription [6, 20]. Incomplete removal of DNA alkylation leads to DNA damage, resulting in cell cycle arrest, inflammation and apoptosis [3–5, 21]. Induction of DNA alkylation by chemotherapeutic agents is a common strategy in cancer treatment to prevent cancer cells from dividing and proliferating [22]. Enzymes that facilitate DNA alkylation damage repair, such as ALKBH2 and ALKBH3, can contribute to resistance to this treatment and insights into their molecular function could provide the basis for developing more efficient cancer therapies [23].

Recently, we described the cooperativity of ALKBH3 and the ASCC3 DNA helicase complex to promote DNA alkylation damage repair in various cancer cells. ALKBH3 knockdown causes elevated levels of 3-meC accompanied by increased DNA damage response (DDR) and reduced cell proliferation [24]. However, the mechanisms of *in vivo* genomic targeting of ALKBH3 are not yet fully understood.

Herein, using chromatin immunoprecipitation experiments followed by massively parallel sequencing analysis (ChIP-seq) we find that in PC3 prostate cancer cells ALKBH3 binding is enriched at transcription associated genomic loci, where ssDNA is accessible. Specifically, we find ALKBH3 bound at active gene promoters, enhancers, and regions with putative quadruplex DNA. Unexpectedly, ALKBH3 binds strongly to the initiation sites of some particularly highly expressed gene promoters. Interestingly, these promoters are bound by an unusually large number of transcriptional regulators, indicating a highly regulated ‘hyperactive’ promoter class. However, we find that loss of ALKBH3 does not directly affect expression of ALKBH3 occupied genes, suggesting a transcription unrelated function of ALKBH3. Instead, upon ALKBH3 knockdown we observe an increased expression of genes involved in inflammatory pathways, which could be a downstream effect of elevated DNA damage after ALKBH3 depletion [24, 25]. The genomic localization of ALKBH3 at transcription-related loci raises the possibility that ALKBH3 could have a role in suppressing transcription-associated DNA damage to preserve the genomic integrity.

## Methods

### Cell culture and viral transduction

U2OS, 293 T, NCI-H23, and PC3 cells were obtained from the American Type Culture Collection (ATCC) and maintained as previously described [24]. ShRNAs constructs, preparation of viruses and cell transduction have been described previously [24]. Cells infected with lentiviral shRNAs were selected after infection with puromycin (1 μg/mL) for at least 48 h.

### Antibodies

Rabbit anti-ALKBH3 antibodies were obtained from Millipore (Catalog #09-882).

### Immunofluorescence (IF)

U2OS and PC3 cells were used for IF and were maintained as described previously [24]. The cells were infected with the indicated lentiviral shRNAs and plated onto coverslips. Cells were then fixed with PBS (ph 7.4) containing 3.2 % paraform for 20 min, washed extensively with IF wash buffer (1X PBS containing 0.5 % NP-40 and 0.02 % NaN_3_), then incubated with blocking buffer (IF wash buffer with 10 % fetal bovine serum), and finally stained with anti-rabbit-ALKBH3 (Millipore) diluted in blocking buffer. Secondary antibodies (goat anti-rabbit Alexa Fluor 488) were from Millipore.

### RNAi experiments, microarray analysis, and qRT-PCR

Lentiviral shRNA against ALKBH3, ALKBH2, and GFP as control were used as described before [24]. Briefly, lentiviral production was carried out in 293 T cells and target cells were infected for 48 h followed by selection with puromycin for 48 h or 96 h. RNA extracted from ALKBH3 knockdown and control cells were sent to Transcriptome Analysis Laboratory (TAL, University Medical Center, Göttingen) for expression analysis. Microarrays were done using the ‘Low RNA Input linear Amplification Kit Plus, One Color’ protocol (Agilent Technologies, Cat. No.: 5188–5339) and the Agilent RNA Spike-In Kit for One color (Agilent Technologies, Cat. No.: 5188–5282) following the manufacturer’s standard protocol. Global gene expression analysis was applied using the Human Gene Expression 4x44K v2 Microarray Kit (Agilent Technologies, Cat. No.: G4845A). 200 ng of total RNA from each sample from PC3 cells were used as a starting material to prepare cDNA. The hybridizations were performed for 17 h at 10 rpm and 65 °C in the Hybridization Oven (Agilent). Washing and staining of the arrays were done according to the manufacturer’s recommendation. Cy3 intensities were detected by one-color scanning using an Agilent DNA microarray scanner (G2505B). Intensity data were extracted using Agilent’s Feature Extraction (FE) software (version 10.5.3.1) including a quality control based on internal controls using Agilent’s protocol GE1_107_Sep09. A subset of genes from microarray results was verified by qRT-PCR (Fig. 3d). For RT-PCR, cells were infected with lentivirus shRNA against ALKBH3 or GFP and selected with puromycin. qRT-PCR was performed using the One-Step Sybr No Rox Kit from Bioline on a CFX384 Real-Time System (Bio-Rad). Primers are shown in Table S1 (Additional file 1).

### ChIP and ChIP-Seq

Chromatin was prepared from PC3 cells as previously described [26], except that it was sonicated to 200 base pairs (bp). Chromatin was incubated with total of 6 μg specific antibodies against ALKBH3 (Millipore) or IgG (Abcam) overnight at 4 °C and then mixed with 50 % slurry protein A beads (Millipore) for 1 h at 4 °C. The beads were washed extensively, de-cross-linked at 65 °C, and treated with RNase A and proteinase K. Samples were next subjected to phenol-chloroform extraction and precipitated with ice-cold ethanol. Libraries were constructed of 50 ng of ChIP’d DNA following Illumina’s® protocol and sequenced using an Illumina Genome Analyzer before being further analyzed bioinformatically. Analysis of DNA via qRT-PCR was performed as described above, with gene-specific primers (Additional file 1: Table S2).

### Bioinformatic analysis

Microarrays were normalized and analyzed using the limma package for Bioconductor [27, 28]. ChIP-Seq data (Additional file 1: Table S3) were mapped to human genome hg19 using bowtie version 1.0 [29], allowing one mismatch (n = 1) and maximal three possible alignments (m = 3). All subsequent analyses of ChIP-Seq data were performed using the Cistrome platform [30, 31] (Galaxy Code 2014.5.5). If possible, data were also directly uploaded into Cistrome from the GEO database. For Promoter definition, RefSeq genes were downloaded from the UCSC Genome Browser. After removal of duplicates with identical transcription start site, 31,296 promoters, including genes with alternative transcription start sites, were used for analysis. Promoters with weakly bound ALKBH3 were identified using the k-means clustering function within the heatmap feature in Cistrome. Enhancers were defined as overlapping peaks (called by MACS with *P* value 1e-05) of H3K4me1 and H3K27ac (from LNCaP cells), which do not overlap with promoters (−1,000/+1,000). ETS transcription factor bindings sites were called using MACS with a cutoff *P* value of 1e-05. Promoters overlapping with an ETS binding site were considered as ETS factor bound promoters. Predicted G4 DNA sites were downloaded from [32] and converted to hg19. Only sites that are not at promoter or enhancers sites were used for analysis. CpG islands and Transcription factor binding sites (TfbsClusteredV3) were downloaded from the UCSC table browser. A promoter transcription factor binding event has been defined as an overlap of a clustered transcription factor binding site with a promoter site. For TATA-box analysis, conserved transcription factor binding sites (tfbsConsSites) were downloaded from UCSC browser. Conserved TBP bindings sites (V$TBP_01) overlapping with promoter sites were considered as TATA-Box. The counting of the ChIP-Seq tags at each promoter was done using a custom R script for Bioconductor.

### Accession numbers

ChIP-Seq and Microarray data are available at the GEO repository with the accession numbers GSE57568 and GSE57591.

### Statistical analyses

The significance of the data was either calculated by Cistrome, via unpaired Student’s *t*-tests, or has been evaluated using hypergeometric probability tests.

## Results

### ALKBH3 occupies ubiquitously expressed promoters in PC3 cells

**Fig. 1:**
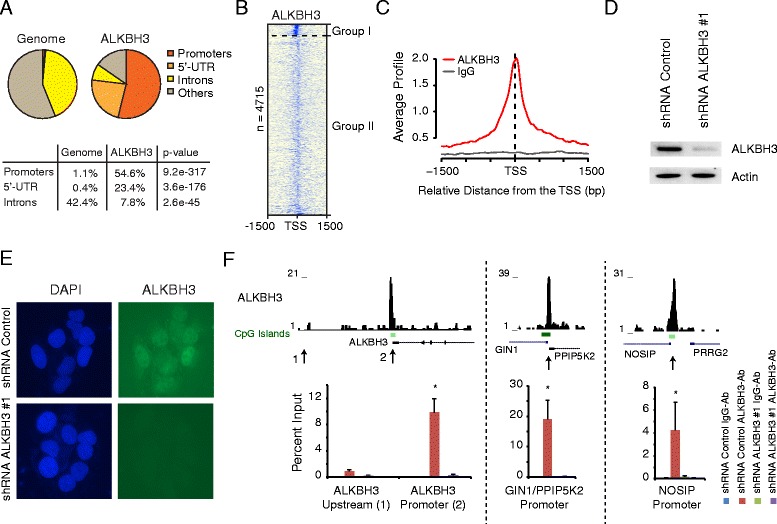
ALKBH3 localizes genome-wide at promoters. **a** High confident (MACS-called) ALKBH3 ChIP-seq peaks are enriched at promoters but are depleted from inter- and intragenic regions. **b** Heatmap of all (including non-MACS called) ALKBH3 bound promoters. Two major groups of ALKBH3 promoters (group I and group II), with substantial different binding properties, have been identified (see also Fig. 4a). **c** ALKBH3 profile at all bound promoters in comparison to ChIP-Seq with control IgG. **d** ALKBH3 shRNA #1 infected PC3 cells have reduced protein levels of ALKBH3, compared to control cells. **e** Immunofluorescence of ALKBH3 in U2OS cells infected with control shRNA or ALKBH3 shRNA #1 demonstrating the specificity of the ALKBH3 antibody. **f** High confidence peaks at three example genes (including the *ALKBH3* gene itself). The ChIP-Seq results have been validated by ChIP qRT-PCR. The enrichment is strongly reduced in the knockdown cells. (* = *P* <0.05). See also Additional file 1: Figure S1

Our previous work showed that ALKBH3 depletion in PC3 prostate cancer cells increases global 3-meC levels and induces H2A.X phosphorylation as well as 53BP1 foci formation [24], demonstrating the occurrence of systemic DNA damage. To gain further genome-wide insights into the DNA repair function of ALKBH3, we performed ChIP-seq experiments in PC3 cells using specific anti-ALKBH3 antibodies. First, we performed Model-based Analysis for ChIP-Seq (MACS) (cutoff *P* value: 1e-04) within the Cistrome platform [30, 31] to identify 423 high confidence ALKBH3 binding sites. ALKBH3 predominantly occupies promoters and regions downstream of the transcription start site (TSS) (5′-UTR), but is excluded from introns and other genomic regions (Fig. 1a). Out of the 423 peaks, 354 are present at known promoters. Further analysis using Cistrome revealed that ALKBH3 is weakly bound but enriched at more than 4,000 additional promoters. We segregated ALKBH3 bound promoters into two groups (I and II) depending on the ALKBH3 binding strength (Figs. 1b, 4a). At these promoters ALKBH3 localizes around the TSS (Fig. 1c). We confirmed the specificity of the antibody via immunofluorescence and ChIP experiments in ALKBH3 knockdown cells (Fig. 1d-f, and Additional file 1: Figure S1a and b). Interestingly, the *ALKBH3* gene promoter is bound by the ALKBH3 protein (Fig. 1f, left panel) and belongs to the promoter group I with strong ALKBH3 enrichment.

**Fig. 2:**
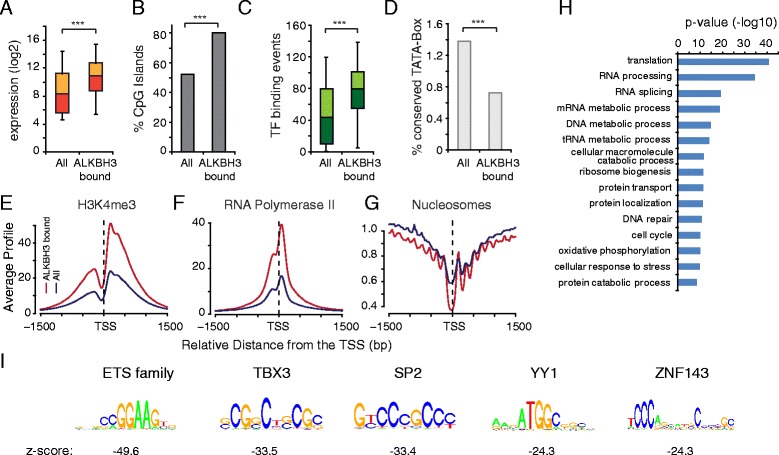
ALKBH3 bound promoters have characteristics of ubiquitously expressed gene promoters. ALKBH3 bound promoters have high expression levels (**a**), often a CpG island (**b**), higher numbers of bound transcription factors (**c**), reduced occurrence of the TATA-Box (**d**), high levels of H3K4me3 (**e**), and RNA polymerase II (**f**) as well as highly order nucleosome positioning (**g**). **h** Gene ontology shows enrichment of general cellular processes. **i** The most strongly enriched transcription factor motifs found at ALKBH3 bound promoters. Significance in (a) and (c) has been calculated via an unpaired Student’s *t*-test. In (b) and (d) the significance has been evaluated using a hypergeometric probability test. The whisker-box plots represent the lower quartile, median and upper quartile of the data with 5 % and 95 % whiskers. TF = Transcription Factor. (*** = *P* <0.001)

In order to examine the general properties of ALKBH3 bound promoters, we compared ALKBH3 binding with publicly available datasets for histone marks, bound proteins, and other features (Additional file 1: Table S3). To examine ALKBH3’s role in cancer we preferentially used data from prostate (for example, LNCaP) or other human cancer cell lines. ALKBH3 bound promoters have elevated gene expression (Fig. 2a), are enriched for CpG islands (Fig. 2b), are bound by multiple transcription factors (TFs) (Fig. 2c), and are enriched for the active histone mark H3K4me3 (Fig. 2e) and RNA Polymerase II (Fig. 2f). In addition, ALKBH3 bound promoters are depleted for the TATA-Box and have highly ordered nucleosome positioning (Fig. 2d and g). These properties are typically found at ubiquitously expressed, house-keeping promoters [33] and gene ontology analysis using DAVID [34] confirmed a strong enrichment of genes involved in general cellular processes, such as translation, RNA processing, and cell metabolism (Fig. 2h). A motif search identified the ETS transcription factor binding motif to be most significantly enriched (Fig. 2i), suggesting that genes occupied by ALKBH3 are possibly activated by one or several ETS factors (Additional file 1: Figure S2), which are often over-expressed in prostate cancer [35, 36]. However, other TFs binding motifs are highly enriched as well (Fig. 2i), supporting the general conclusion that ALKBH3 bound promoters are strongly regulated and transcriptionally active.

### Depletion of ALKBH3 induces an inflammatory response

**Fig. 3:**
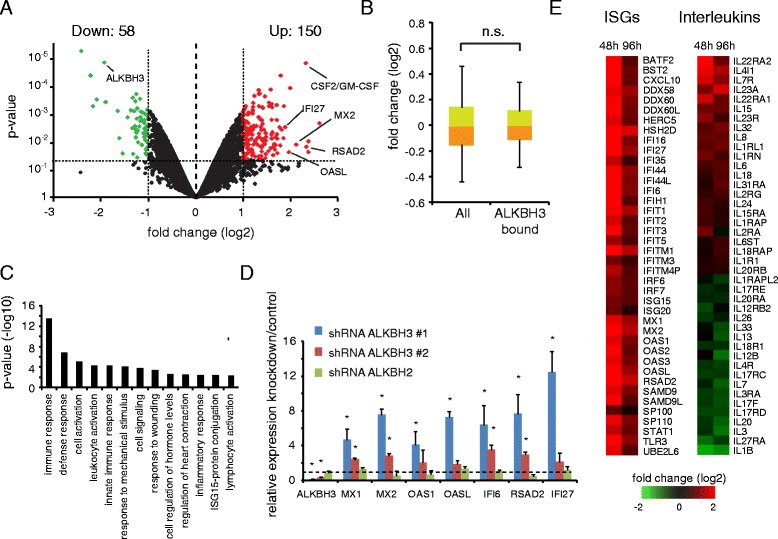
ALKBH3 depletion induces an inflammatory response. **a** Volcano plot of the microarray data after ALKBH3 knockdown. A two-fold expression change and a *P* value <0.05 were used as cutoff. **b** Gene expression does not significantly change at ALKBH3 target genes. **c** Gene ontology analysis of upregulated genes showed strong induction of inflammatory genes. **d** qRT-PCR analysis of several interferon stimulated genes (ISGs) with two independent ALKBH3 shRNAs in comparison to ALKBH2 shRNA infected cells. The data were normalized via GAPDH to control shRNA infected cells. Error bars show standard deviation of triplicates. Significance was tested using a student’s *t*-test. *P* <0.05 has been considered as significant. **e** Heatmaps showing the expression change of affected interferon inducible genes (ISGs) and interleukins, at 48 h and 96 h post selection

Previous work for ALKBH1, ALKBH2, and ALKBH4 suggested that oxidative DNA demethylases could be directly involved in gene regulation [37–39]. To address if ALKBH3 affects target gene transcription, we knocked down ALKBH3 and extracted mRNA for microarray analysis at two different time points after selection (48 h and 96 h). We consistently identified 150 upregulated and 58 downregulated genes (Fig. 3a). However, unlike ALKBH1 [39] or ALKBH2 [38] depletion, genes bound by ALKBH3 did not show any significant expression level changes upon ALKBH3 knockdown (Fig. 3b).

To gain deeper insight into which genes are affected by ALKBH3 knockdown, we performed gene ontology (GO) analysis of the upregulated genes and found that many genes are involved in the innate/inflammatory immune response (Fig. 3a, c, and e). We confirmed, by qRT-PCR, the specific upregulation of several inflammatory genes after ALKBH3 but not ALKBH2 knockdown (Fig. 3d). Interferon stimulated genes (ISGs) are strongest induced at 48 h, but their expression declines at 96 h after ALKBH3 knockdown, indicating a negative feedback loop could exist (Fig. 3e).

Since most inflammatory genes are not directly bound by ALKBH3, we speculate that the inflammatory response may be an indirect consequence of DNA damage accumulation in the ALKBH3 deficient cells [24], because DNA damage triggers the activation of inflammatory and other pathways [3–5, 25, 40]. Interestingly, a similar phenomenon has been described in HeLa cells for the knockdown of the ALKBH3 interacting [24] ASCC3 DNA helicase [41]. ALKBH3 knockdown also causes an upregulation of inflammatory genes in the non-small cell lung adenocarcinoma cell line NCI-H23 (Additional file 1: Figure S1c), suggesting that ablation of ALKBH3-dependent DNA repair mechanisms induces inflammatory pathways in multiple cancer cell lines.

Together these data suggest that ALKBH3 does not function to regulate transcriptional activity. Instead, the induction of inflammatory genes supports a potential role of ALKBH3 at its genomic targets to remove DNA alkylation adducts, such as 3-meC or 1-meA [16, 18, 19, 24], in order to prevent DNA damage.

### ALKBH3 occupies places with ssDNA

**Fig. 4:**
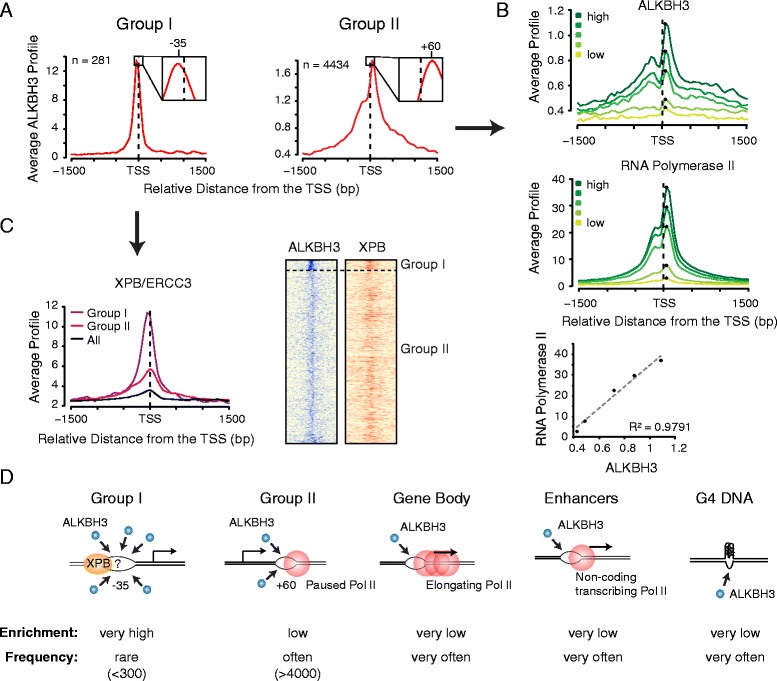
ALKBH3 is recruited to locations with single-stranded DNA. **a** ALKBH3 profiles at the two groups of ALKBH3 bound promoters. Group I is characterized by a strong binding upstream of the transcription start site. Group II shows a weaker binding downstream of the TSS and a ‘shoulder’ upstream of the TSS. **b** Profiles of ALKBH3 and RNA polymerase II at of all promoters (excluding group I promoters) divided in quintiles according their gene expression. ALKBH3 and RNA polymerase II profiles show an almost linear correlation, supporting interdependency. **c** Group I promoters are characterized by a strong occupancy of the TFIIH helicase XPB/ERCC3 (data from HT1080 cells). The profile of XPB at group I promoters culminates upstream of the transcription start site, similar to ALKBH3. The heatmaps show the comparison of ALKBH3 (identical to Fig. 1b) and XPB at all ALKBH3 bound promoters (−1,500/+1,500 bp). **d** Overview of putative ALKBH3 targets in PC3 cells and the respective ALKBH3 enrichment and cellular frequency. The left panel shows a speculative formation of an atypical transcription initiation bubble at group I promoters, potentially explaining the strong recruitment of ALKBH3. See also Additional file 1: Figure S4

Next we wished to determine if ALKBH3 was bound to specific DNA regions. Previous work showed that for DNA demethylation ALKBH3 prefers to demethylate ssDNA over dsDNA substrates [16, 18, 19]. This raises the possibility that for its DNA repair function, ALKBH3 is recruited to genomic places with ssDNA. At promoters, ssDNA is created during transcription initiation and at paused RNA polymerases II complexes. We asked whether ALKBH3 occupancy could be associated with those features. The ALKBH3 binding profile of group I promoters is characterized by a very strong enrichment slightly upstream of the transcription start site, while the group II promoter profile peaks mainly downstream of the TSS and shows only a moderate ALKBH3 binding level (Fig. 4a). Since the two distinct positions and binding strengths might reflect different ALKBH3 targets, we analyzed these two groups further separately. Interestingly, group I promoters have higher transcriptional activity compared to group II promoters (Additional file 1: Figure S3e and f).

The group II promoter profile is similar to the RNA polymerase II profile - both peak at +60 bp and possess a ‘shoulder’ upstream of the transcription start site (Fig. 4a and b). This correlation suggests that ALKBH3 recruitment could be dependent on the formation of single-stranded transcription bubbles at places with paused RNA polymerase II. To investigate this possibility, we divided all promoters (excluding group I promoters) into five different classes according their transcription levels. RNA polymerase II occupancy and ALKBH3 binding showed an almost linear correlation (Fig. 4b). These findings are consistent with the idea that ALKBH3 might get recruited to the transcription bubble of paused RNA polymerase II, analogous to what has been hypothesized before for AID (Activation Induced Cytidine Deaminase) [42].

Interestingly, the ALKBH3 group I promoter profile does not overlap significantly with RNA polymerase II. Instead, the ALKBH3 binding profile culminates slightly upstream of the TSS, at the site of transcription initiation (Fig. 4a). We speculated that at group I promoters ALKBH3 might target the single stranded DNA bubble established upon transcription initiation. DNA unwinding and formation of ssDNA during initiation depends on the DNA helicase XPB [43]. Investigation of published ChIP-Seq data for XPB from HT1080 cells [44] revealed that XPB also has stronger binding at group I relative to group II promoters. Furthermore, XPB is enriched upstream of the TSS at group I but not at group II promoters, similar to ALKBH3 (Fig. 4c). These observations imply a stronger activity of XPB at the initiation site of group I promoters, which may lead to an elevated occurrence of ssDNA. We hypothesize that this increased ssDNA level might be the basis for the increased ALKBH3 recruitment. Notably, HT1080 cells are fibrosarcoma cells, but despite the different origin of PC3 and HT1080 cells, we still see this correlation of ALKBH3 and XPB binding. We reasoned that this promoter type may be present in a broad range of cell types, and we therefore analyzed these promoters bioinformatically using additional datasets (see below).

Single-stranded DNA occurs not only at promoters, but also at other places in the genome. During transcription elongation, the transcription bubble moves along the gene. Interestingly, highly expressed genes, which have elevated transcription elongation, have elevated ALKBH3 levels in the gene body (Additional file 1: Figure S4a). We also observed a mild ALKBH3 enrichment at enhancers where RNA polymerase II transcription has been reported [45] (Additional file 1: Figure S4b and d). Recently the role of G-quadruplex DNA (G4 DNA) during genome-wide regulatory processes was described [46]. G4 DNA contains an accumulation of guanines that leads to the formation of a stable DNA quadruplex and the occurrence of ssDNA, in particular on the reverse strand [46]. We found ALKBH3 is mildly enriched at places with potential G4 DNA (Additional file 1: Figure S4c and d).

Together these findings support the hypothesis that ALKBH3 is recruited to places in the genome with accessible ssDNA (summarized in Fig. 4d), in agreement with ALKBH3’s repair function at ssDNA [6, 16, 18, 19].

### Group I promoters are a putative novel hyperactive promoter class

**Fig. 5:**
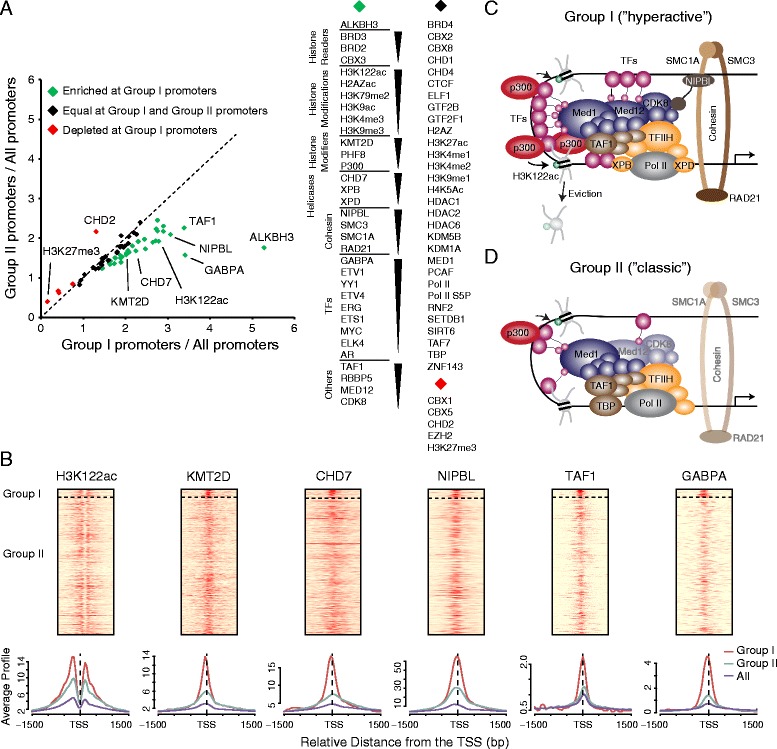
Activating factors are enriched at group I promoters. **a** Multiple factors were investigated to characterize group I promoters. For each feature the number of ChIP-Seq tags was counted per promoter (see Additional file 1: Figure S5). The mean tag number was used to examine whether or not a factor is enriched/depleted at group I promoters relative to group II promoters. X = Mean of group I promoters/Mean of all promoter. Y = Mean of group II promoters/Mean of all promoter. Many factors are stronger enriched at group I promoters than at group II promoters (at least 10 % more, green). **b** Six examples of highly enriched factors. The promoters in the heatmaps are sorted as in Fig. 1b (−1,500/+1,500 bp). The average profiles show a strong enrichment at group I promoters. **c** Hypothetical model of the group I promoter functioning. At group I promoters, transcription factors (purple) are clustered, which correlates with the presence of the Mediator complex and cohesin. P300 is recruited by transcription factors and Mediator and might facilitate H3K122 acetylation, which could lead to nucleosome eviction. Mediator (blue), together with TFIID (brown) and TFIIH (yellow) and possibly other factors might form a non-classical initiation complex, leading to stronger transcriptional activity. **d** At group II promoters the levels of regulatory factors is less, leading to classical initiation processes

The binding of ALKBH3 at the transcription initiation site of group I promoters (Fig. 4a) prompted us to examine this promoter group in more detail. Interestingly, comparison of group I and group II promoters revealed differences in TF binding frequency (Additional file 1: Figure S3h). We found that group II promoters have on average 75 TF binding events while group I promoters have on average 112 TF binding events (50 % more). ETS transcription factors display a more pronounced difference (80 % more) (Additional file 1: Figure S2c). This indicates a clustering of transcription factors at these promoters, which is commonly associated with cohesin, the Mediator complex and other features [47, 48]. To further characterize the group I promoters, we performed a comprehensive analysis of numerous transcription associated features (Fig. 5a, Additional file 1: Figure S5). A large proportion of the investigated features are enriched at group I relative to group II promoters, indicating non-typical regulation (Fig. 5a). TAF1, a major subunit of TFIID, is one the most enriched factor (50 % more). In contrast, TBP (TATA-Box binding protein), another subunit of TFIID, is not significantly enriched, suggesting that at those promoters a TBP-independent recruitment of TFIID may occur more often than on other promoters [49]. Further, the helicase CHD7, but not CHD1, CHD2, and CHD4, is strongly enriched (30 % more), raising the possibility that CHD7 could play a role in unwinding the DNA, in addition to XPB. Most active histone marks are mildly enriched, while the repressive histone mark H3K27me3 and its methyltransferase EZH2 are depleted. Interestingly, the histone modification with the strongest increase at group I promoters is H3K122 acetylation (40 % more), which has been demonstrated to enhance nucleosome eviction (Fig. 5a and b) [50]. Since H3K122 acetylation is mediated by the histone acetyltransferase p300, and we also found a significant increase of p300, we speculate that acetylation of H3K122 by p300 and subsequent histone eviction could be of particular importance at these promoters [50]. This idea is further supported by a reduced nucleosome occupancy and increased DNase I hypersensitivity at the initiation site of group I promoters (Additional file 1: Figure S3c and d). In addition to histone acetyltransferases, enzymes that regulate histone methylation, such as KMT2D (deposits H3K4me3) and PHF8 (removes H3K9me1/2, H3K27me2, and H4K20me1) are enriched as well (Fig. 5a and b).

Taken together, these data suggest that numerous activating factors are highly enriched at group I promoters, indicating a massive regulation, which consequently leads to higher transcriptional activity (‘hyperactive’) (Fig. 5c and d; Additional file 1: Figure S3e and f). A list of genes with this promoter type in PC3 cells is presented in Table S4 (Additional file 1). The presence of ALKBH3 at some of those gene promoters was confirmed in NCI-H23 lung cancer cells (Additional file 1: Figure S1d), suggesting that the genomic binding pattern of ALKBH3 as well as the occurrence of hyperactive promoters is similar in ALKBH3 over-expressing cancer cells.

## Discussion

Previous work suggested a pivotal role of ALKBH3 in multiple cancer types, such as prostate [24, 51], pancreatic [52], urothelial [53], non-small-cell lung [54], papillary thyroid [55], and brain [56] cancer. However, the cellular role of ALKBH3 is not yet fully understood. In order to gain further insights into the function of ALKBH3 particular in cancer, we applied genome wide approaches using PC3 prostate cancer cells as a model.

We initially performed genome localization studies to identify genomic regions bound by ALKBH3. We found that ALKBH3 preferentially occupies locations where ssDNA is occurring, such as promoters, enhancers, and G4 DNA (Fig. 4). This finding suggests that ALKBH3 is directed to ssDNA regions. In the future it will be exciting to determine whether this correlation is reflective of a regulated process and what the precise mechanism for ALKBH3 recruitment could be. Does this recruitment depend on ssDNA formation?

ALKBH3 was most enriched at the initiation site of a small number of highly expressed genes (Fig. 4a). Further investigation of these promoters led to the hypothesis that they are a putative novel ‘hyperactive’ subgroup of ubiquitously expressed gene promoters (Fig. 5).

Characterization of the ALKBH3 bound promoters was carried out using publicly available data from different research groups and different cell types. Despite use of this relatively heterogeneous dataset, we were still able to detect a correlation between strong ALKBH3 binding at group I promoters and enrichment of factors involved in gene activation. This study highlights how publicly available datasets can be utilized to develop novel hypotheses, while creating these data *ab initio* would neither be timely nor financially feasible.

Since we see this correlation of highest ALKBH3 binding with enriched binding of other factors across multiple cancer cell types we conclude that these hyperactive promoters exist in a relatively cell type independent manner. However, we cannot exclude that a transition between group I and group II promoters can occur and that a group II promoter might get promoted to a hyperactive promoter, and vice versa, once the level of activating factors at the promoter exceeds or falls below a certain threshold, respectively. Since we hardly see promoters where the ALKBH3 binding is in an intermediate state between group I and group II promoters, we hypothesize that these transitions – if they take place – are rare. It also remains to be determined whether these hyperactive promoters are restricted to highly proliferative cancer cells or if they occur in all cell types.

ALKBH3 binding to group I promoters is about eight-fold stronger than to group II promoters. We hypothesize that an elevated formation or an increased accessibility of ssDNA at the initiation site of group I promoters is the source for the increased ALKBH3 binding (Fig. 4d, left panel). We speculate that an enhanced presence of DNA helicases, such as the TFIIH helicases XPB and XPD, CHD7, or the ALKBH3 interacting ASCC3 helicase could lead to increased exposed ssDNA and therefore facilitate ALKBH3 recruitment [24, 57, 58]. The formation of ssDNA could also rely on bending of DNA upon binding of transcription factors, such as ETS factors and YY1 [59, 60]. It is also possible that group I promoters do not have increased accessible ssDNA and that the recruitment of ALKBH3 is facilitated by some unknown mechanism that does not depend on ssDNA.

We performed microarray analysis to elucidate whether ALKBH3 affects transcription of its target genes (Fig. 3). We observed no significant changes of the transcription of the ALKBH3 bound genes, suggesting that ALKBH3 binding does not directly regulate transcription. However, since ALKBH3 bound genes are highly expressed, subtle changes caused by ALKBH3 depletion might not be detected via microarray. Thus, our results do not explicitly exclude the possibility that ALKBH3 plays a similar expression regulating role as described for ALKBH1 and ALKBH2 [38, 39].

**Fig. 6:**
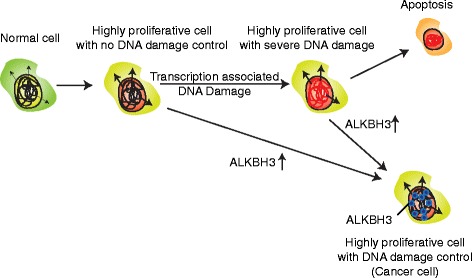
Model of potential role of ALKBH3 in cancer. A normal cell might alter in a way that results in increased gene expression (for example, via upregulation of ETS transcription factors [36] (Additional file 1: Figure S2b)) and therewith increases the proliferative capacity of the cell. The increased transcription level elevates the global amount of accessible ssDNA, which is attacked by DNA damaging agents. Uncontrolled this leads to accumulation of DNA damage and apoptosis. After upregulation of ALKBH3 for example due to binding of ETS factors to the *ALKBH3* promoter (Additional file 1: Figure S2d) and its potential transition to a hyperactive promoter (Fig. 1f, left panel) the DNA damage might become under control and the cells can continuously sustain a high proliferation rate

Knockdown of ALKBH3 in PC3 cells leads to an induction of inflammatory response gene expression (Fig. 3), which might be a consequence of elevated 3-meC levels and DNA damage after ALKBH3 depletion [3, 4, 24, 25, 61]. Most cancer cells, including PC3 cells, have rapid proliferation and accordingly elevated transcriptional activity [62]. One consequence of elevated transcription is increased sensitivity of DNA for DNA alkylation damage, including 3-meC [6, 63–65]. The ALKBH3 genomic binding profile suggests that ALKBH3 is an intrinsic DNA repair protein that removes DNA alkylation that might occur naturally during transcription [63, 66]. ALKBH3 upregulation in cancer could be an important step during cancerogenesis to achieve an increased proliferation rate while maintaining genomic integrity (Fig. 6). If true, this would suggest that ALKBH3 inhibition could potentially slow cancer progression [67].

Since ALKBH3 demethylates RNA in addition to ssDNA [16, 18], it is possible that ALKBH3 could also have a role in the demethylation of primary RNA transcripts at its genomic targets [6]. Whether this could be of physiological relevance will be of interest to be determined in the future.

## Conclusions

Alkylating agents that methylate DNA and disrupt genomic integrity of fast proliferating cells are widely used in cancer treatment [22]. Due to its localization at transcription-associated loci, we suggest that ALKBH3 is an intrinsic DNA repair protein that suppresses transcription associated DNA alkylation damage at highly expressed genes. The over-expression of ALKBH3 in cancer cells might facilitate alkylation damage resistance during cancer treatment and therefore raises the possibility of ALKBH3 as a potential anticancer target in the future [23, 67].

The genome-wide binding pattern of ALKBH3 revealed a strong binding to the initiation sites of a small number of highly active promoters. We hypothesize that these promoters are a new class of ubiquitously expressed promoters, which may have a specific initiation process, allowing ALKBH3 to ‘mark’ these promoters. Follow-up investigation of those hyperactive promoters *in vitro*, *in vivo*, and via bioinformatics will help to better understand their mechanisms as well as their role in transcription regulation and during cancer progression.

## Additional file

Additional file 1:
**Tables S1 to S4 and Figures S1 to S5. Table S1.** List of the used qRT-PCR primers for cDNA. **Table S2.** List of the used qRT-PCR primers for ChIP. **Table S3.** List of public data used for bioinformatics analysis [44, 47, 50, 68–82]. **Table S4.** List of hyperactive promoters in PC3 cells. **Figure S1.** ALKBH3 Immunofluorescence in PC3 cells, ChIP at additional ALKBH3 targets in PC3 cells, induction of inflammatory genes in NCI-H23 cells, ALKBH3 ChIP in NCI-H23 cells. **Figure S2.** Bioinformatic analysis of relationship of ETS transcription factors, ALKBH3 and gene expression. **Figure S3.** A more detailed analysis of promoters such as in Fig. 2. **Figure S4.** Shows enrichment of ALKBH3 in the gene body, at enhancers and places of G4 DNA. **Figure S5.** Shows whisker blots of the counted ChIP-Seq tags at promoters from all analyzed features in Fig. 5a.
