# Supplementary material for: The oxidative demethylase ALKBH3 marks hyperactive gene promoters in human cancer cells
Source: Genome Med. 2015 Jun 30;7(1):66. doi: 10.1186/s13073-015-0180-0 (PMC4517488; doi:10.1186/s13073-015-0180-0)
Supplement: Additional file 1: — Tables S1 to S4 and Figures S1 to S5. Table S1. List of the used qRT-PCR primers for cDNA. Table S2. List of the used qRT-PCR primers for ChIP. Table S3. List of public data used for bioinformatics analysis [44, 47, 50, 68–82]. Table S4. List of hyperactive promoters in PC3 cells. Figure S1. ALKBH3 Immunofluorescence in PC3 cells, ChIP at additional ALKBH3 targets in PC3 cells, induction of inflammatory genes in NCI-H23 cells, ALKBH3 ChIP in NCI-H23 cells. Figure S2. Bioinformatic analysis of relationship of ETS transcription factors, ALKBH3 and gene expression. Figure S3. A more detailed analysis of promoters such as in Fig. 2. Figure S4. Shows enrichment of ALKBH3 in the gene body, at enhancers and places of G4 DNA. Figure S5. Shows whisker blots of the counted ChIP-Seq tags at promoters from all analyzed features in Fig. 5a. [file 13073_2015_180_MOESM1_ESM.pdf]

Table S1: qPCR Primer for cDNA

GAPDH f	GGAGCGAGATCCCTCCAAAAT
GAPDH r	GGCTGTTGTCATACTTCTCATGG
ALKBH3 f	CATGGAAGGAGCGACACAAGC
ALKBH3 r	CTAGAGTGGTATTCTTTGGGC
MX1 f	GTTTCCGAAGTGGACATCGCA
MX1 r	CTGCACAGGTTGTTCTCAGC
MX2 f	CAGAGGCAGCGGAATCGTAA
MX2 r	TGAAGCTCTAGCTCGGTGTTC
OAS1 f	TGTCCAAGGTGGTAAAGGGTG
OAS1 r	CCGGCGATTTAACTGATCCTG
OASL f	CTGATGCAGGAACTGTATAGCAC
OASL r	CACAGCGTCTAGCACCTCTT
IFI6 f	GGTCTGCGATCCTGAATGGG
IFI6 r	TCACTATCGAGATACTTGTGGGT
IFI27 f	TGCTCTCACCTCATCAGCAGT
IFI27 r	CACAACTCCTCCAATCACAACT
RSAD2 f	TGGGTGCTTACACCTGCTG
RSAD2 r	GAAGTGATAGTTGACGCTGGTT
DCP1A f	GAATGACTGTCACCGCATAGC
DCP1A r	CTGAGTGCTTGGCTGTAACCC
RPS25 f	AAGGACGCTGGAAAGTCGG
RPS25 r	CTTTGCCTTTGGACCACTTCT
TATDN3 f	GTAGGCTTGGTGGACTGTCAC
TATDN3 r	CCACAAGGGCCACAACATTG
OTUD7B f	GTCAGATTTTGTCCGTTCCACA
OTUD7B r	CATGGACTTGACGTAGCTGTT
BRF2 f	GGTGGAAGACTCGCACTATTC
BRF2 r	CGACTAACTTGTTCGTTTTCCCC
DVL2 f	GAGGAAGAGACTCCCTACCTG
DVL2 r	CGGGCGTTGTCATCTGAAAT
TMEM126A f	TAACACGGAGTGGACTGACTG
TMEM126A r	CTTTGTGTGGTAACAGAGCTGAT
BLID f	TACACAGGATGGATAGAGCGAG
BLID r	CTTTGTTGGAACCCAAGAGCG
FBXO22 f	CGGAGCACCTTCGTGTTGA
FBXO22 r	CACACACTCCCTCCATAAGCG
STARD9 f	AGGTGGACAATCGACCAGATG
STARD9 r	CCAGTAGCAGTAATCAAAGCCAA
ANP32A f	CACCTCAATCGCAAACTTACCA
ANP32A r	AACACATTTTCTCGGTAGTCGTT
CACNA1G f	TGTCTCCGCACGGTCTGTAA
CACNA1G r	AAGCCGGTTCCAAGTGTCTC
NCF4 f	CGGGCCGAGAGTGACTTTG
NCF4 r	TCTTCACCTCGATGACGAAAAC
FECH f	GGAGATGTTCACGACTTCCTTC
FECH r	GAATGGTGCCAGCTTATTCTGA
DBIL5P2 f	CTGAAGGGTACTGTGTGCGAT
DBIL5P2 r	CATGCCTCCTACTTGGCCT

Table S2: qPCR Primer for ChIP

ALKBH3 upstr f	CAAGAGCTGCCTACCAAAGG
ALKBH3 upstr r	TACTGTTGGCCTGTGCAGAA
ALKBH3 f	TATCCCCTCATCAGCACCTT
ALKBH3 r	GACTCCAGCAACTCCCAATC
NOSIP f	ATCCGGAACTTTCGATTAGC
NOSIP r	CAGAAGGCACCGGAAGTAGA
GIN1/PPIP5K2 f	ACACAGCACACCAGAACGAC
GIN1/PPIP5K2 r	CTTTCTGACCGTGGTTCCAA
ANAPC5 f	GAGCTCCTCAGTCCCTTCAG
ANAPC5 r	TGTCAGAGCACATGGGAGAG
ADAT2/PEX3 f	AGCGTAGCTGCTTTGCTGTA
ADAT2/PEX3 r	CGTCCCGTTCTGTGCTCT
GAPDH f	CAATTCCCCATCTCAGTCGT
GAPDH r	TAGTAGCCGGGCCCTACTTT

Table S3: Datasets used for bioinformatics analysis

Factor	ID	Cell Type	Reference
ALKBH3	GSM1385219	PC3	this study
IgG	GSM1385220	PC3	this study
AR	GSM1328945/GSM1328947	VCaP	[1]
BRD2	GSM971946	HEK293	[2]
BRD3	GSM971947	HEK293	[2]
BRD4	GSM1328959	VCaP	[1]
CBX1	GSM971950	HEK293	[2]
CBX2	GSM1003567	K562	[3]
CBX3	GSM1003568	K562	[3]
CBX5	GSM971949	HEK293	[2]
CBX8	GSM1003569	K562	[3]
CDK8	GSM937561	HCT116	[4]
CHD1	GSM1003575	K562	[3]
CHD2	GSM935502	K562	[3]
CHD4	GSM1003510	K562	[3]
CHD7	GSM1003478	K562	[3]
CTCF	GSM822311	K562	[3]
DNase I	GSM822387	LNCaP	[5]
ELF1	GSM1424527	DU145	[6]
ELK4	GSM1424528	DU145	[6]
ERG	GSM353648	LNCaP	[7]
ETS1	GSM738821	RWPE1	[8]
ETV1	GSM1145322	LNCaP	[9]
ETV4	GSM738817	PC3	[8]
EZH2	GSM969570	LNCaP	[10]
GABPA	GSM738822	RWPE1	[8]
GTF2B	GSM935394	K562	[3]
GTF2F1	GSM935501	K562	[3]
H2A.Z	GSM686941	LNCaP	[11]
H2A.Zac	GSM1059388	MCF7	[12]
H3K4me1	GSM353634	LNCaP	[7]
H3K4me2	GSM353635	LNCaP	[7]
H3K4me3	GSM353626	LNCaP	[7]
H3K9Ac	GSM733778	K562	[3]
H3K9me1	GSM733777	K562	[3]
H3K9me3	GSM353625	LNCaP	[7]
H3K27ac	GSM686937	LNCaP	[11]
H3K27me3	GSM969571	LNCaP	[10]
H3K79me2	GSM733653	K562	[3]
H3K122Ac	GSM1059385	MCF7	[12]
H4K5Ac	GSM686939	LNCaP	[11]
HDAC1	GSM1003448	K562	[3]
HDAC2	GSM1003447	K562	[3]
HDAC6	GSM1003504	K562	[3]
KDM5B	GSM1003586	K562	[3]
KDM1A	GSM1003570	K562	[3]
MED1	GSM894081	H2171	[13]
MED12	GSM686945	LNCaP	[11]
KMT2D	GSM1240109	HCT116	[14]
Myc	GSM822310	K562	[3]
NIPBL	GSM1208661	LoVo	[15]
Nucleosomes	wgEncodeEH000922	GM12878	[16]
PCAF	GSM831007	K562	[3]
P300	GSM686943	LNCaP	[11]
PHF8	GSM1003450	K562	[3]
RAD21	GSM1208668	Lovo	[15]
RBBP5	GSM1003449	K562	[3]
RNA Polymerase II	GSM699636	LNCaP	[17]
RNA Polymerase II S5P	GSM831016	K562	[3]
RNF2	GSM1003563	K562	[3]
SETDB1	GSM1003452	K562	[3]
SIRT6	GSM1003560	K562	[3]
SMC1A	GSM1208678	LoVo	[15]
SMC3	GSM1208679	LoVo	[15]
TAF1	GSM803431	K562	[3]
TAF7	GSM803407	K562	[3]
TBP	GSM935495	K562	[3]
XPB	GSM1092544	HT1080	[18]
XPD	GSM1092545	HT1080	[18]
YY1	GSM803354	HCT116	[3]
ZNF143	GSM959047	K562	[3]

1. Asangani IA, Dommeti VL, Wang X, Malik R, Cieslik M, Yang R, Escara-Wilke J, Wilder-Romans K, Dhanireddy S, Engelke C, et al: **Therapeutic targeting of BET bromodomain proteins in castration-resistant prostate cancer.** *Nature* 2014, **510:**278-282.
2. LeRoy G, Chepelev I, DiMaggio PA, Blanco MA, Zee BM, Zhao K, Garcia BA: **Proteogenomic characterization and mapping of nucleosomes decoded by Brd and HP1 proteins.** *Genome Biol* 2012, **13:**R68.
3. ENCODE Project Consortium: **An integrated encyclopedia of DNA elements in the human genome.** *Nature* 2012, **489:**57-74.
4. Galbraith MD, Allen MA, Bensard CL, Wang X, Schwinn MK, Qin B, Long HW, Daniels DL, Hahn WC, Dowell RD, Espinosa JM: **HIF1A employs CDK8-mediator to stimulate RNAPII elongation in response to hypoxia.** *Cell* 2013, **153:**1327-1339.
5. He HH, Meyer CA, Chen MW, Jordan VC, Brown M, Liu XS: **Differential DNase I hypersensitivity reveals factor-dependent chromatin dynamics.** *Genome Res* 2012, **22:**1015-1025.
6. Plotnik JP, Budka JA, Ferris MW, Hollenhorst PC: **ETS1 is a genome-wide effector of RAS/ERK signaling in epithelial cells.** *Nucleic Acids Res* 2014, **42:**11928-11940.
7. Yu J, Mani RS, Cao Q, Brenner CJ, Cao X, Wang X, Wu L, Li J, Hu M, Gong Y, et al: **An integrated network of androgen receptor, polycomb, and TMPRSS2-ERG gene fusions in prostate cancer progression.** *Cancer Cell* 2010, **17:**443-454.
8. Hollenhorst PC, Ferris MW, Hull MA, Chae H, Kim S, Graves BJ: **Oncogenic ETS proteins mimic activated RAS/MAPK signaling in prostate cells.** *Genes Dev* 2011, **25:**2147-2157.
9. Chen Y, Chi P, Rockowitz S, Iaquinta PJ, Shamu T, Shukla S, Gao D, Sirota I, Carver BS, Wongvipat J, et al: **ETS factors reprogram the androgen receptor cistrome and prime prostate tumorigenesis in response to PTEN loss.** *Nat Med* 2013, **19:**1023-1029.
10. Xu K, Wu ZJ, Groner AC, He HH, Cai C, Lis RT, Wu X, Stack EC, Loda M, Liu T, et al: **EZH2 oncogenic activity in castration-resistant prostate cancer cells is Polycomb-independent.** *Science* 2012, **338:**1465-1469.
11. Wang D, Garcia-Bassets I, Benner C, Li W, Su X, Zhou Y, Qiu J, Liu W, Kaikkonen MU, Ohgi KA, et al: **Reprogramming transcription by distinct classes of enhancers functionally defined by eRNA.** *Nature* 2011, **474:**390-394.
12. Tropberger P, Pott S, Keller C, Kamieniarz-Gdula K, Caron M, Richter F, Li G, Mittler G, Liu ET, Buhler M, et al: **Regulation of transcription through acetylation of H3K122 on the lateral surface of the histone octamer.** *Cell* 2013, **152:**859-872.
13. Lin CY, Loven J, Rahl PB, Paranal RM, Burge CB, Bradner JE, Lee TI, Young RA: **Transcriptional amplification in tumor cells with elevated c-Myc.** *Cell* 2012, **151:**56-67.
14. Hu D, Gao X, Morgan MA, Herz HM, Smith ER, Shilatifard A: **The MLL3/MLL4 branches of the COMPASS family function as major histone H3K4 monomethylases at enhancers.** *Mol Cell Biol* 2013, **33:**4745-4754.
15. Yan J, Enge M, Whitington T, Dave K, Liu J, Sur I, Schmierer B, Jolma A, Kivioja T, Taipale M, Taipale J: **Transcription factor binding in human cells occurs in dense clusters formed around cohesin anchor sites.** *Cell* 2013, **154:**801-813.
16. Kundaje A, Kyriazopoulou-Panagiotopoulou S, Libbrecht M, Smith CL, Raha D, Winters EE, Johnson SM, Snyder M, Batzoglou S, Sidow A: **Ubiquitous heterogeneity and asymmetry of the chromatin environment at regulatory elements.** *Genome Res* 2012, **22:**1735-1747.
17. Tan PY, Chang CW, Chng KR, Wansa KD, Sung WK, Cheung E: **Integration of regulatory networks by NKX3-1 promotes androgen-dependent prostate cancer survival.** *Mol Cell Biol* 2012, **32:**399-414.
18. Gray LT, Vallur AC, Eddy J, Maizels N: **G quadruplexes are genomewide targets of transcriptional helicases XPB and XPD.** *Nat Chem Biol* 2014.

Table S4: Genes with hyperactive promoters in PC3 cells

AAR2	chr20
ACOT13	chr6
ACP2	chr11
ADAT2	chr6
ADPRHL1	chr13
AHSA1	chr14
ALG10B	chr12
ALG3	chr3
ALKBH3	chr11
ANAPC5	chr12
ANKRD40	chr17
AQR	chr15
ARHGAP1	chr11
ASAH2B	chr10
AURKAIP1	chr1
BBS1	chr11
BMS1	chr10
BMS1P4	chr10
BRF2	chr8
C8orf33	chr8
C11orf31	chr11
C11orf49	chr11
C11orf57	chr11
C11orf74	chr11
C12orf73	chr12
C12orf76	chr12
C17orf75	chr17
C18orf21	chr18
C19orf52	chr19
C19orf82	chr19
C21orf59	chr21
C21orf67	chr21
CCAR2	chr8
CDC16	chr13
CDK5RAP1	chr20
CENPP	chr9
CGGBP1	chr3
COMMD2	chr3
COPS2	chr15
COPS4	chr4
COPS7B	chr2
COX16	chr14
CPSF1	chr8
CSTF2T	chr10
DCAF10	chr9
DCDC1	chr11
DCP1A	chr3
DDX55	chr12
DHX33	chr17
DMAP1	chr1
DNAJC2	chr7
DNAJC24	chr11
DNAJC25	chr9
DPAGT1	chr11
DPM3	chr1
DPY19L4	chr8
DRG2	chr17
DSCR3	chr21
DSTYK	chr1
DTWD1	chr15
ECD	chr10
EFCAB7	chr1
EIF1AD	chr11
EIF3F	chr11
EIF4E2	chr2
EMC4	chr15
EMG1	chr12
ERH	chr14
EXD2	chr14
EXOSC4	chr8
FAM126B	chr2
FAM149B1	chr10
FAM207A	chr21
FAM227B	chr15
FAM98B	chr15
FBXO28	chr1
FBXW11	chr5
FEM1B	chr15
FRA10AC1	chr10
GABPA	chr21
GALK2	chr15
GIN1	chr5
GOLGA3	chr12
GRPEL2	chr5
GSTCD	chr4
GTF2H2	chr5
GTF2H2B	chr5
GTF2H2C	chr5
GTF2H2D	chr5
GTF3C3	chr2
GTPBP3	chr19
HDAC8	chrX
HELQ	chr4
HEMK1	chr3
HMGXB3	chr5
HNRNPA1L2	chr13
HUS1	chr7
ICT1	chr17
IGHMBP2	chr11
INO80B	chr2
INTS12	chr4
INTS5	chr11
IPO4	chr14
ITGB3BP	chr1
JMJD4	chr1
JPX	chrX
JRK	chr8
KBTBD4	chr11
KCTD10	chr12
KCTD5	chr16
KIAA0391	chr14
KLHL20	chr1
KRR1	chr12
LINC00657	chr20
LINC00910	chr17
LOC100133315	chr11
LOC100288974	chr10
LOC652276	chr16
LSG1	chr3
MAN2C1	chr15
MED18	chr1
MED23	chr6
METTL15	chr11
MITD1	chr2
MKRN2	chr3
MNAT1	chr14
MPLKIP	chr7
MRPL1	chr4
MRPL13	chr8
MRPL16	chr11
MRPL21	chr11
MRPL3	chr3
MRPL30	chr2
MRPL39	chr21
MRPL44	chr2
MRPL48	chr11
MRPS18C	chr4
MRPS31	chr13
MRPS31P5	chr13
MTBP	chr8
MTHFD2L	chr4
MTIF2	chr2
MTRF1	chr13
NDUFA12	chr12
NDUFAF1	chr15
NDUFB3	chr2
NDUFS3	chr11
NDUFS7	chr19
NFKBIL1	chr6
NIF3L1	chr2
NKAP	chrX
NKAPP1	chrX
NME1	chr17
NMNAT1	chr1
NOL12	chr22
NOSIP	chr19
NR1H2	chr19
NR1H3	chr11
NSL1	chr1
NUF2	chr1
NUP107	chr12
OPA1	chr3
OTUD7B	chr1
PCYT1A	chr3
PEX3	chr6
PIGL	chr17
PIH1D2	chr11
PLEKHM3	chr2
POLR3B	chr12
PPIP5K2	chr5
PSMF1	chr20
PTRH2	chr17
RARS	chr5
RBBP5	chr1
RBM25	chr14
RBM28	chr7
RFXANK	chr19
RGS5	chr1
RP11-608O21.1	chr4
RPL29	chr3
RPL37	chr5
RPS18	chr6
RPS25	chr11
RPS27	chr1
RPS7	chr2
RRP15	chr1
RTTN	chr18
SAR1B	chr5
SBDSP1	chr7
SEC13	chr3
SEC22B	chr1
SENP1	chr12
SF3A3	chr1
SF3B14	chr2
SLC33A1	chr3
SLC39A9	chr14
SMG7-AS1	chr1
SMG8	chr17
SNHG6	chr8
SNRNP27	chr2
SNRPB	chr20
SPAG8	chr9
SREK1IP1	chr5
SSBP1	chr7
STX16	chr20
STX18	chr4
SUPT7L	chr2
TACO1	chr17
TARS2	chr1
TATDN3	chr1
TBC1D19	chr4
TDP2	chr6
TEFM	chr17
THAP10	chr15
THEM4	chr1
TIAL1	chr10
TIGD1	chr2
TIGD6	chr5
TIMM9	chr14
TMEM101	chr17
TMEM128	chr4
TMEM222	chr1
TMEM242	chr6
TMEM41A	chr3
TMEM69	chr1
TOP3B	chr22
TRAPPC4	chr11
TRIP4	chr15
TTC26	chr7
TTC4	chr1
TUBGCP3	chr13
TUT1	chr11
TVP23B	chr17
TXNDC9	chr2
UBA5	chr3
UBE3B	chr12
USP30	chr12
UTP3	chr4
VPS25	chr17
VPS45	chr1
VPS51	chr11
VPS52	chr6
VWA9	chr15
WDR11	chr10
WDR24	chr16
WDR31	chr9
WDR36	chr5
YBEY	chr21
YIPF2	chr19
ZBTB11	chr3
ZKSCAN4	chr6
ZMPSTE24	chr1
ZNF205	chr16
ZNF248	chr10
ZNF35	chr3
ZNF408	chr11
ZNF461	chr19
ZNF490	chr19
ZNF579	chr19
ZNF581	chr19
ZNF646	chr16
ZNF668	chr16
ZNF770	chr15
ZNF791	chr19
ZNF846	chr19
ZSCAN9	chr6

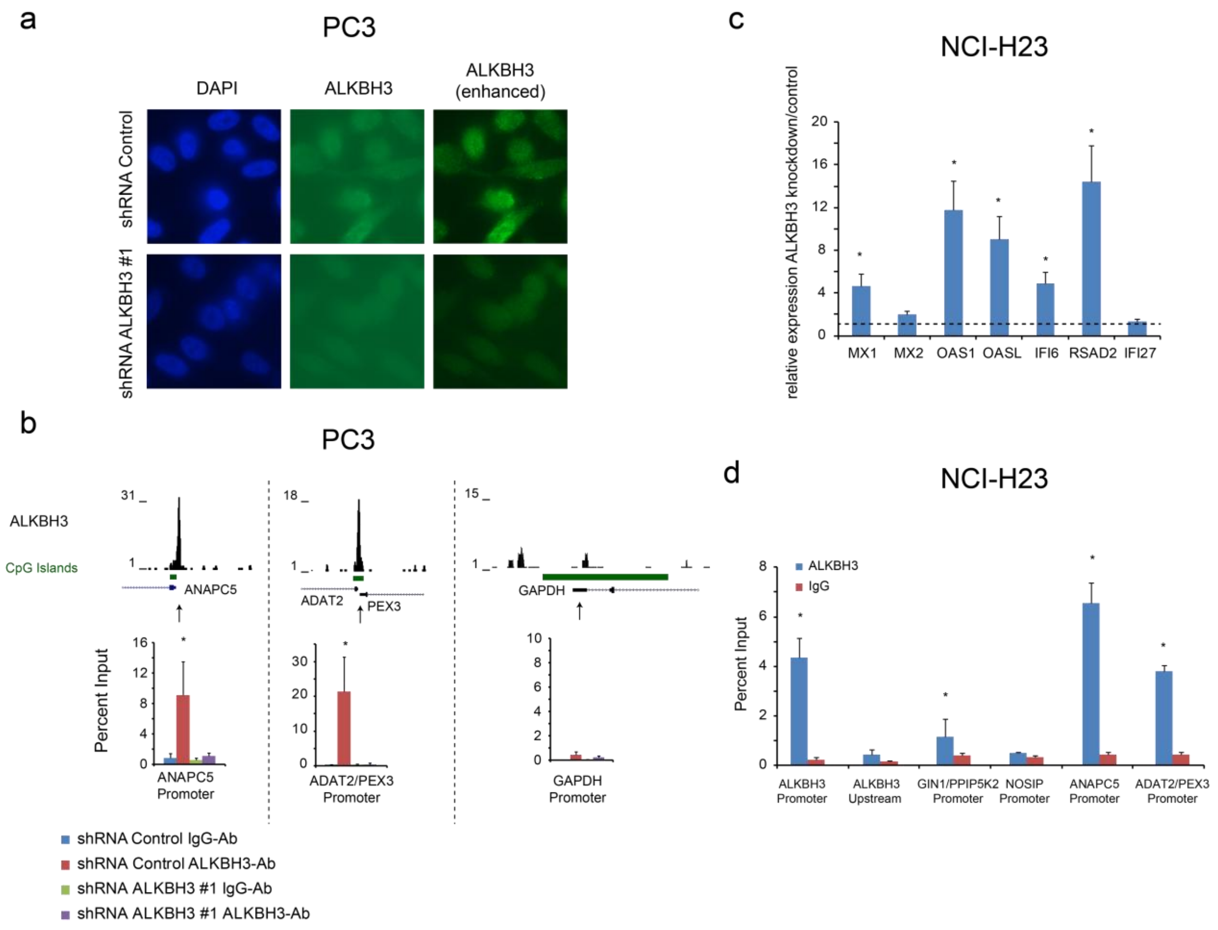

Figure S1: Additional control experiments in PC3 and NCI-H23 cells.
a) Immunofluorescence using ALKBH3 antibody in PC3 cells expressing an ALKBH3 shRNA or a control shRNA. The ALKBH3 immunofluorescence signal was enhanced in the right panel. **b)** ChIP experiment in PC3 cells measured on 2 additional ALKBH3 targets (see Figure 1F) and the GAPDH promoter as negative control. **c)** Quantitative expression analysis of inflammatory genes upon knockdown of ALKBH3 in NCI-N23 lung cancer cells. Data were normalized to GAPDH. **d)** ALKBH3 ChIP in NCI-H23 cells show enrichment of ALKBH3 at some of its targets, found in PC3 cells.

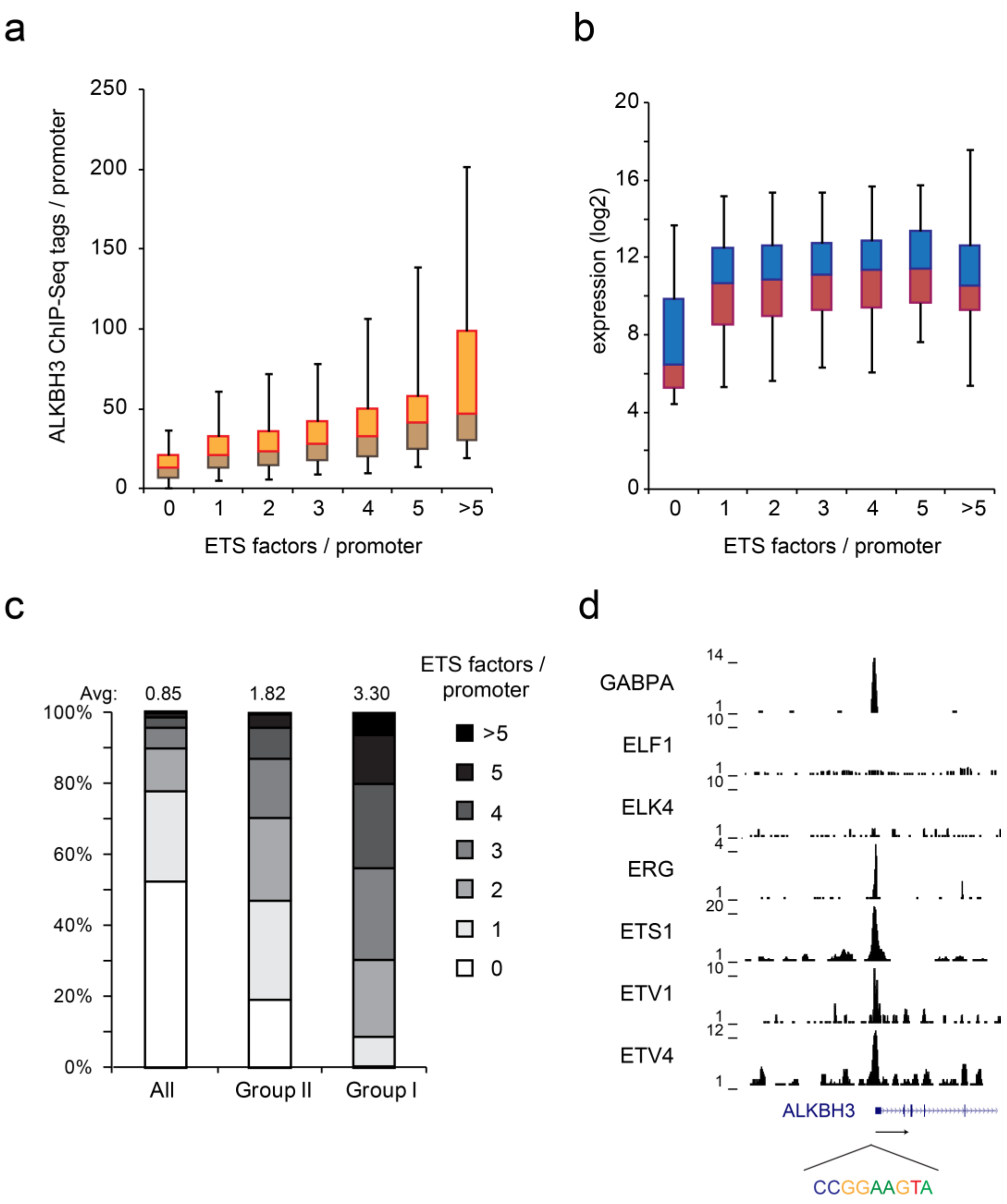

Figure S2: ALKBH3 promoter binding and gene expression correlates with presence of ETS transcription factors.

a-c) Promoters were categorized based on the presence (called by MACS with p-value 1e-05) of seven ETS transcription factors (GABPA, ELF1, ELK4, ERG, ETS1, ETV1, ETV4) in prostate cancer cells (from “0” to “>5” n = 15815; 8330; 3942; 1968; 853; 309; 64). **a)** The level of ALKBH3 at promoters strongly correlates with the number of ETS transcription factors present. **b)** Gene expression (data from PC3 cells) shows correlation with the presence of ETS transcription factors. Genes with at least one bound ETS transcription factor are significantly more active than genes without ETS factor (p-value = 0), suggesting that upregulation of ETS transcription factors in prostate cancer increases the global gene expression level. **c)** Group I promoters are typically targeted by more ETS factors than other promoters. **d)** In prostate cancer cells, the *ALKBH3* gene promoter is occupied by at least five ETS transcription factors. The *ALKBH3* gene promoter possesses an ETS transcription factor binding motif 90 bp upstream from the TSS.

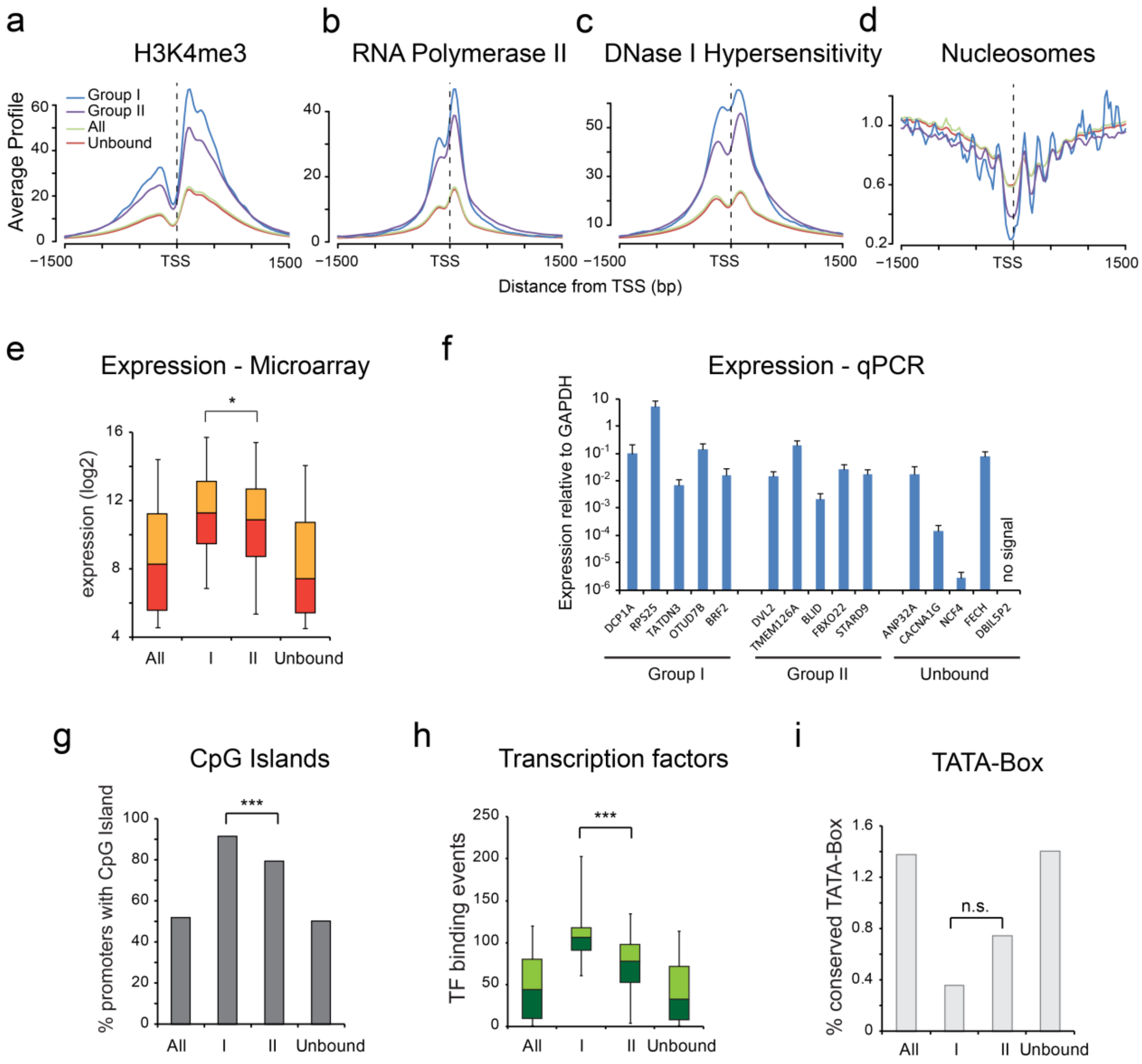

Figure S3: Detailed analysis of ALKBH3 bound promoter with comparison of group I and group II promoters.

a-e;g-i) Same Analysis as in Figure 2, but here the two groups of ALKBH3 bound promoters were investigated separately and ALKBH3-unbound promoters were added as additional group. Group I promoters are generally in a more active state compared to group II promoters. **f)** Five genes were randomly selected from each promoter group and expression levels in PC3 cells were quantitatively measured by qPCR. The results show a similar trend as the microarray data.

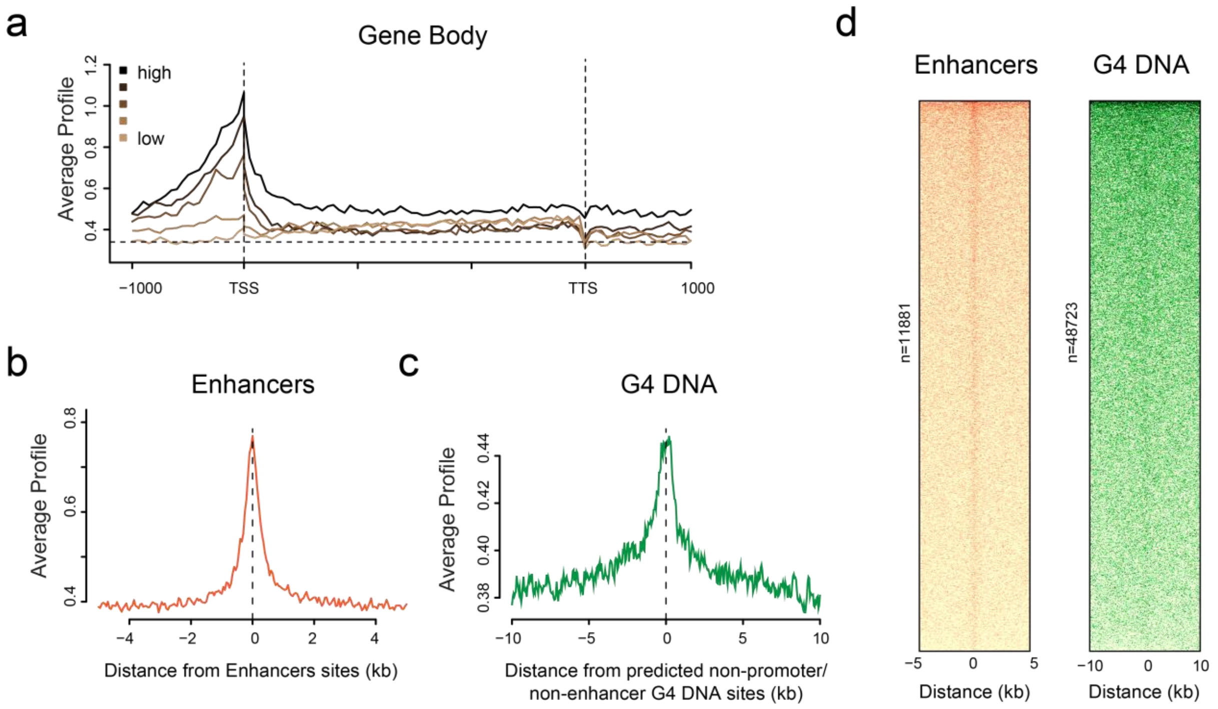

Figure S4: ALKBH3 is genome wide enriched at gene bodies, enhancers and location of G4 DNA.

a) All genes were grouped in quintiles based on the gene expression. ALKBH3 is enriched within gene bodies of highly expressed genes. **b-d)** ALKBH3 is enriched at enhancer sites and sites with putative quadruplex DNA. The heatmaps in (d) were sorted after mean intensity.

Figure S5: Whisker blots for analyzed features.

For each investigated feature presented in Figure 5a the ChIP-Seq tags were counted for each individual promoter (-1000/+1000). The whisker blots represents the distribution of those tags in each promoter group (from left: all promoters, Group II promoters, Group I promoters) with the lower quartile, median and upper quartile of the data and with 5% and 95% whiskers. The red line connects the mean tag number. The number in the plots indicates the p-value for a student's t-test between group I and group II.

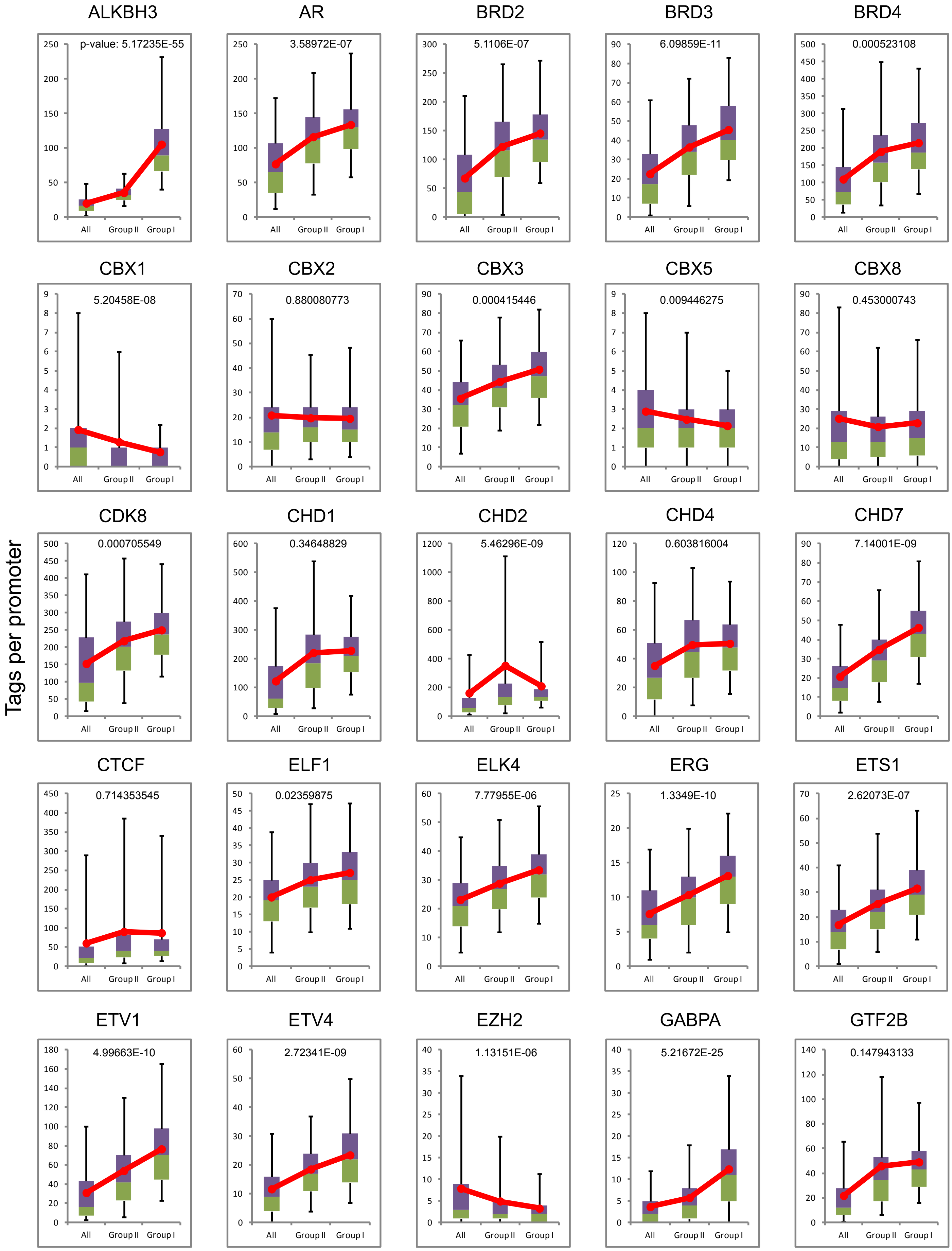

Tags per promoter
ALKBH3
p-value: 5.17235E-55
AR
3.58972E-07
BRD2
5.1106E-07
BRD3
6.09859E-11
BRD4
0.000523108
CBX1
5.20458E-08
CBX2
0.880080773
CBX3
0.000415446
CBX5
0.009446275
CBX8
0.453000743
CDK8
0.000705549
CHD1
0.34648829
CHD2
5.46296E-09
CHD4
0.603816004
CHD7
7.14001E-09
CTCF
0.714353545
ELF1
0.02359875
ELK4
7.77955E-06
ERG
1.3349E-10
ETS1
2.62073E-07
ETV1
4.99663E-10
ETV4
2.72341E-09
EZH2
1.13151E-06
GABPA
5.21672E-25
GTF2B
0.147943133
All
Group II
Group I

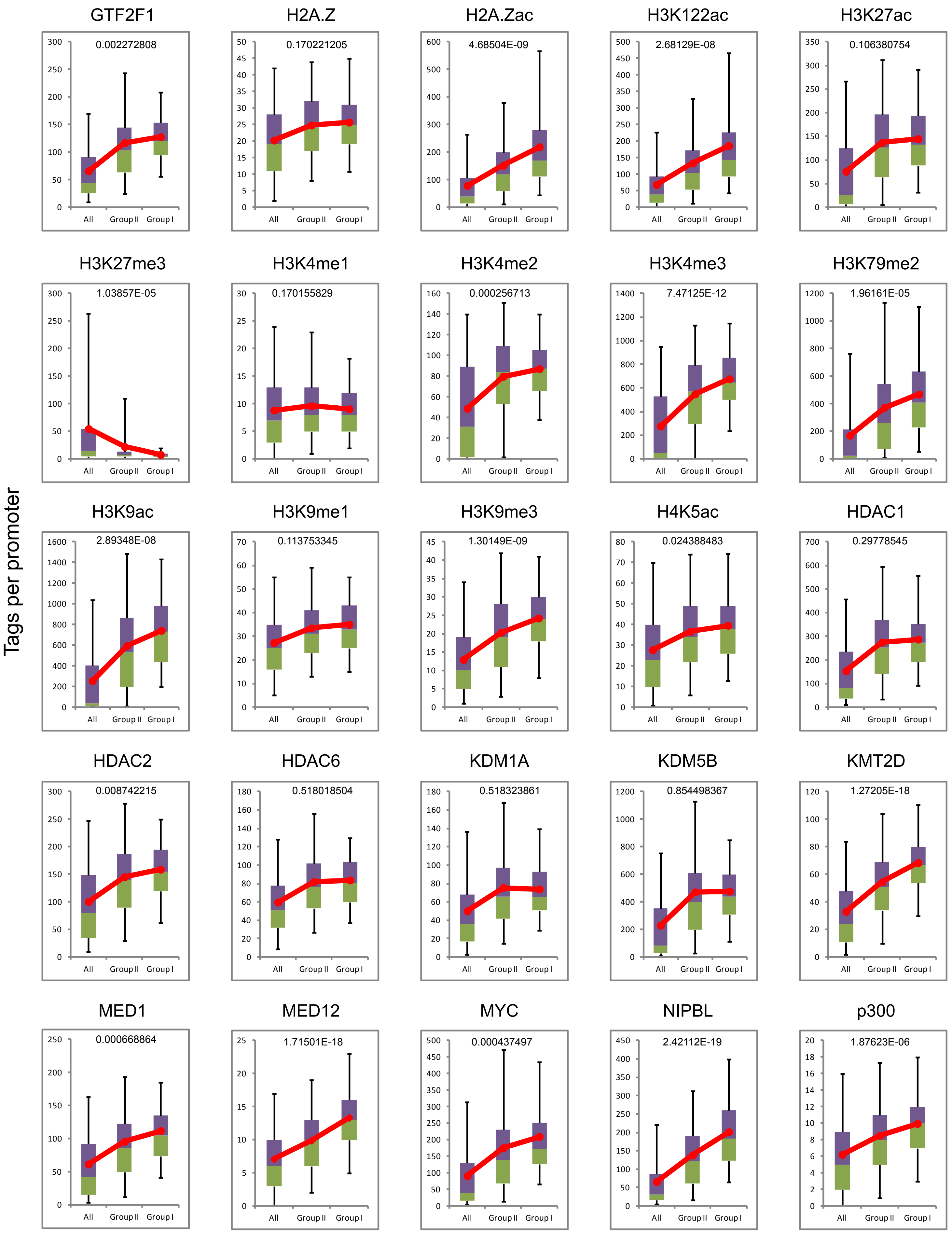
Tags per promoter
GTF2F1
0.002272808
H2A.Z
0.170221205
H2A.Zac
4.68504E-09
H3K122ac
2.68129E-08
H3K27ac
0.106380754
H3K27me3
1.03857E-05
H3K4me1
0.170155829
H3K4me2
0.000256713
H3K4me3
7.47125E-12
H3K79me2
1.96161E-05
H3K9ac
2.89348E-08
H3K9me1
0.113753345
H3K9me3
1.30149E-09
H4K5ac
0.024388483
HDAC1
0.29778545
HDAC2
0.008742215
HDAC6
0.518018504
KDM1A
0.518323861
KDM5B
0.854498367
KMT2D
1.27205E-18
MED1
0.000668864
MED12
1.71501E-18
MYC
0.000437497
NIPBL
2.42112E-19
p300
1.87623E-06
All
Group II
Group I

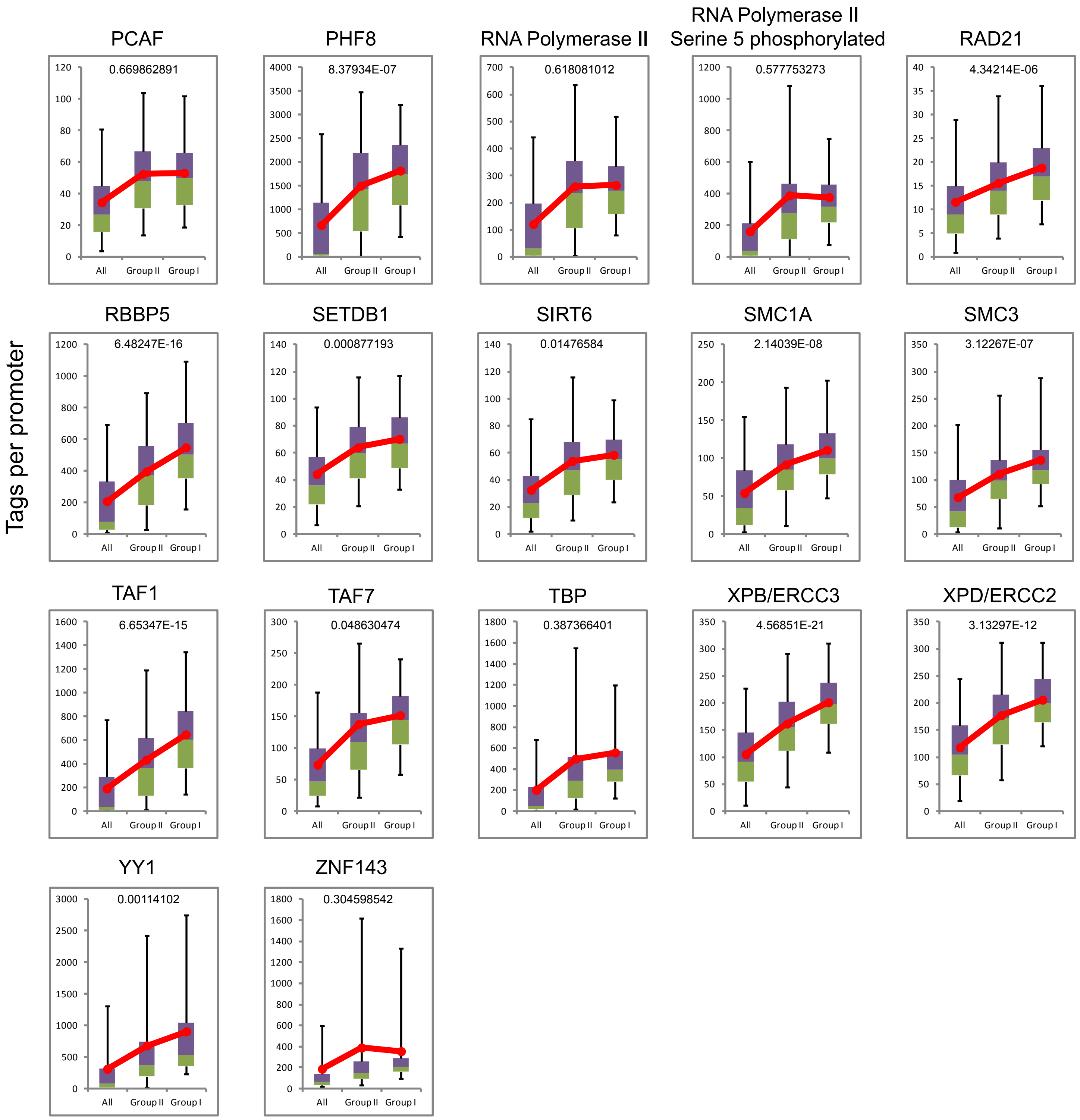
Tags per promoter
PCAF
0.669862891
PHF8
8.37934E-07
RNA Polymerase II
0.618081012
RNA Polymerase II
Serine 5 phosphorylated
0.577753273
RAD21
4.34214E-06
RBBP5
6.48247E-16
SETDB1
0.000877193
SIRT6
0.01476584
SMC1A
2.14039E-08
SMC3
3.12267E-07
TAF1
6.65347E-15
TAF7
0.048630474
TBP
0.387366401
XPB/ERCC3
4.56851E-21
XPD/ERCC2
3.13297E-12
YY1
0.00114102
ZNF143
0.304598542
All
Group II
Group I
